# Facebook Groups for the Management of Chronic Diseases

**DOI:** 10.2196/jmir.7558

**Published:** 2018-01-17

**Authors:** Stephanie R Partridge, Patrick Gallagher, Becky Freeman, Robyn Gallagher

**Affiliations:** ^1^ Prevention Research Collaboration Sydney School of Public Health, Charles Perkins Centre The University of Sydney Sydney Australia; ^2^ Sydney Nursing School Charles Perkins Centre The University of Sydney Sydney Australia

**Keywords:** social media, prevention, intervention, Facebook

## Abstract

The use of Facebook groups by health care researchers and professionals for chronic disease management, namely type 2 diabetes mellitus and coronary heart disease, is in its early stages and challenges are emerging. While Facebook groups offer great potential to deliver health support, research of Facebook groups for chronic disease management remains in its infancy, with robust evidence not yet available. Designing Facebook groups that are acceptable to users, health care researchers as well as health care professionals is a challenge, and there is a poor fit with traditional research and evaluation methods. Key recommendations for future research of Facebook groups for chronic disease management include: (1) iterative content development with input from the target patient population; (2) further understanding of the potential role of group “champions”; (3) ensuring the social media policies of health care institutions allow for real time online communication; and (4) utilizing comprehensive evaluation strategies, including the use of process evaluations.

## Introduction

Patient education is a core component of chronic disease self-management, and is particularly important for type 2 diabetes mellitus (T2DM) [1] and coronary heart disease (CHD) care [2]. Group-based education provides opportunities for the delivery of detailed information, patient discussions, peer support and direct supervision, and support for behaviors such as exercise [1,2]. There is substantial evidence highlighting the benefits of peer support programs in regard to changing behaviors and reducing risk factors [1,2]. Despite their proven effectiveness, the logistics and costs of staffing and providing the specialized venues for in-person, group-based programs ultimately limits accessibility because services must be offered at fixed and limited times and locations [3]. For instance, attendance at traditional cardiac rehabilitation group-based programs and T2DM group-based self-education is persistently low, at 30% and 48% of those referred, respectively [4,5].

Online, real-time social media platforms, such as Facebook, may offer solutions to existing problems with accessing traditional group-based programs for chronic disease management. In mid-2017, Facebook’s community reached two billion people [6]. The continued rise in users is partly due to the growing number of older adults (>65 years) who are joining the social networking site [7]. Recent data suggests that nearly 90% of older adults who were on Facebook reported using the social network to find and share health information [8]. With over 40% of older adults living with two or more chronic conditions [9], the ubiquity of Facebook in their everyday lives has contributed to the emergence of a potential new era of health care information delivery.

Social media interactions enable individuals to read and post material at any time, and from any location, as a part of their usual routine, substantially eliminating obstacles to participation compared to in-person interactions. While expert health care staff are still required, costs may be reduced through more convenient and effective scheduling. However, the potential for Facebook groups to provide novel methods for delivering group-based health care, and enabling support from health care professionals and peers, is yet to be fully harnessed [10].

At this present stage, systematic reviews and meta-analyses are not warranted as previous research has only investigated existing publicly available Facebook groups for general chronic disease management [11], and specifically for T2DM management [12,13], diabetic foot care [14] and hypertension [15]. Encouragingly, two studies are underway investigating the effectiveness of Facebook groups for T2DM [16] and CHD [17]. Other studies with social media groups or features were in young populations or evaluated as a part of larger multicomponent mobile health (mHealth) programs where individual effectiveness of the group could not be determined. Therefore, in this viewpoint, we discuss the issues and potential benefits of using Facebook groups for the management of chronic diseases, namely T2DM and CHD, and provide recommendations for researchers working in this space.

## Facebook Groups for the Management of Chronic Diseases

There is emerging evidence that chronic disease self-management programs delivered by alternative means, such as telehealth and electronic health (eHealth) delivery, have comparable outcomes to in-person programs [18,19]. However, recent reviews have attempted to determine the effectiveness of social media, using evidence arising largely from multicomponent telehealth and eHealth interventions, which includes social media features [20-22]. The difficulty with this lack of demarcation is that social media interventions may be more complex than previously thought and create difficulty for replication and implementation into practice [23,24].

Facebook groups may offer a mutual platform of support for the management of chronic diseases. Facebook defines their group feature as “a space to communicate about shared interests with certain people” [25]. The Facebook group feature allows patients and/or health professionals to interact through posts, which includes writing and responding to posts, in a self-subscribing forum [25]. Groups can either be open, closed or private. Closed or private groups are commonly chosen for health research, as only group members can view the content [25]. Other group features include the capacity to allocate moderating privileges to selected members [25].

Facebook groups can enable health care professionals to give both individuals and groups support, advice, and encouragement to foster self-management and behavior change [26,27]. Further health benefits can result through the development of collective knowledge, social networking, and peer-to-peer information exchange. However, to provide robust evidence for replicatation, there are key issues in the development, implementation and evaluation of Facebook groups that require further research.

## Development, Implementation and Evaluation of Facebook Groups

Developing Facebook groups that are acceptable to and effective for people with chronic disease, as well as health care professionals and researchers, is a challenge requiring engagement of multidisciplinary teams [28]. While there is good evidence about how best to run in-person peer support groups across a variety of health conditions [29], there is limited guidance for how to develop content and effective engagement and communication strategies for Facebook groups to assist people with the management of T2DM or CHD.

Content for in-person chronic disease management peer support groups cannot be directly converted to group-based interventions on Facebook, due to the difference in communication mechanisms. Pagoto et al [30] developed a model for the adaptation of behavioral interventions for social media delivery. This model provides guidance for content conversion and recommends that the content library aligns with how potential users interact with the Facebook platform [30]. An iterative content design process with input from the target audience has the potential to increase appeal and effectiveness of a Facebook group [31-33].

Formative research on older adults with chronic diseases is lacking, with only two studies investigating cardiac patients’ frequency of social media use [34], and experience and perceptions of using Facebook [35]. One study assessed how patients with T2DM communicate health information using Facebook [36]. Research investigating Facebook groups has been predominantly focused on younger people targeting single behaviors [37-54]. Evidence from such interventions is not generalizable to older populations, considering the differences in Facebook use and communication preferences between the two generations [32].

Moderators of Facebook groups need to be aware of the use and communication preference of older adults living with chronic diseases, as well as being experienced Facebook users themselves [30]. Considering the initial complexity of managing T2DM and CHD, initial group moderation by a health professional may be most appropriate. In addition to the moderators, there is a need to have an existing support network prior to participant enrolment, to avoid the “empty room” phenomenon [28]. This can theoretically be achieved by enrolling peer “champions,” whose role is to actively encourage participants to engage with each other [55]. The role and training of these peer “champions” requires greater understanding, as well as the ideal size of a Facebook support group for a chronic disease management.

Moderators may also be required to provide initial education and reminders to inform group members about the privacy settings of the group, as well as their personal account. All posts within a closed or private Facebook group are only visible to moderators and group members. However, issues such as data security and privacy of data management on commercial platforms, like Facebook, requires further attention.

Health care institutions’ policies on the use of social media by health care staff need to be flexible to account for the real-time nature of conversations on Facebook [30]. This is not currently standard practice in many health care institutional policies. For example, some health care institutions require staff, who are representing the institution on Facebook, to have all posts approved by a supervisor one month prior to posting [56]. This hinders not only the continuous and dynamic nature of conversations on Facebook, but also the progression of research in this space.

Analysis of publicly available Facebook groups on chronic disease showed that the majority of groups identified were focused on awareness creation [11,15]. However, the few support groups for patients with chronic disease provided insights about effective content and communication strategies. For example, in the case of T2DM education, Facebook group participation has demonstrated improved knowledge, skills, confidence, and notably improved patient self-management [12]. Higher levels of interaction were seen on posts about peers’ personal experiences and realistic self-depictions of living with a chronic disease [13,14]. The usefulness of some groups was associated with the types of posts, and no association was found with the number of likes or presence of user comments [14]. This shows the potential capacity of Facebook groups to offer indirect support [27], and highlights that engagement cannot always be determined by common Facebook analytics, such as the number of likes and comments per post.

The additional challenge of evaluating Facebook groups is that many types of data, including both quantitative and qualitative, must be collected to assess engagement and effectiveness. If the Facebook group is a component of a multicomponent program, process evaluations can potentially provide insight into the causal mechanisms of such interventions and enable fine-grained understanding of the individual components [57,58]. Process evaluation methodology is underutilized in multicomponent intervention research, and this challenges research translation to identify essential intervention components from those that are not as important [59].

## Conclusions

No robust evidence presently exists to showcase the advantages and/or disadvantages of using chronic disease peer support groups on Facebook. This is partly because only publicly accessible, peer-led groups have been evaluated or groups have only been evaluated as part of larger multicomponent mHealth programs. While Facebook groups can reduce the participant burden of engaging in in-person group support programs, further research is required to understand their potential future role in chronic disease management.

## Abbreviations

CHD: coronary heart disease
eHealth: electronic health
mHealth: mobile health
T2DM: type 2 diabetes mellitus
